# Neuroblastoma Metastasis to the Mandible in Children: A Case Report and Focused Narrative Review of Reported Cases

**DOI:** 10.3390/children13080989

**Published:** 2026-07-25

**Authors:** Ronja Marquardt, Simon Hundeshagen, Felix Tilsen, Frank Tavassol, Waldemar Reich

**Affiliations:** Department of Oral and Maxillofacial Surgery, University Hospital Halle (Saale), Martin Luther University Halle-Wittenberg, 06120 Halle (Saale), Germanyfelix.tilsen@uk-halle.de (F.T.); frank.tavassol@uk-halle.de (F.T.)

**Keywords:** child, mandibular metastasis, mandible, MYCN amplification, neuroblastoma, pediatric oncology, oral and maxillofacial surgery, case report

## Abstract

**Highlights:**

**What are the main findings?**

- Mandibular metastasis of neuroblastoma in children is rare but may be the first clinical manifestation of disseminated disease.
- Reported cases commonly presented with mandibular or facial swelling, intraoral masses, pain, tooth mobility or displacement, and symptoms mimicking odontogenic or inflammatory disease.

**What are the implications of the main findings?**

- Although odontogenic and inflammatory etiologies are more common, malignant disease, including metastatic neuroblastoma and other pediatric tumors, should be considered in the differential diagnosis of atypical jaw or facial swelling.
- Persistent, atypical, rapidly progressive, or therapy-resistant mandibular swelling in infants and young children should prompt early imaging, biopsy, and interdisciplinary evaluation.

**Abstract:**

**Background:** Neuroblastoma is a common extracranial solid malignant tumor of early childhood; however, mandibular involvement is rare and may mimic odontogenic or inflammatory disease. **Case Presentation:** We report an 8-month-old girl with left paramandibular swelling initially suspected to represent parotitis or odontogenic inflammation. Imaging revealed a destructive mandibular lesion with sunburst periosteal reaction, and histology confirmed undifferentiated neuroblastoma. Staging identified a left primary adrenal tumor with extensive bone marrow infiltration and MYCN proto-oncogene amplification. The patient received multimodal high-risk neuroblastoma therapy, including chemotherapy, surgery, autologous stem cell transplantation, proton therapy, antibody therapy, and Lorlatinib. Despite radiological remission, she developed severe pulmonary complications and died shortly before the age of five years. **Methods:** A focused literature review was conducted to identify published pediatric cases of metastatic neuroblastoma involving the mandible. **Results:** Through our review, we identified 31 published pediatric cases of mandibular metastatic neuroblastoma. Reported cases most commonly described mandibular swelling, pain, tooth mobility, and facial asymmetry. Most mandibular lesions represented metastatic disease from an adrenal or abdominal primary tumor. **Conclusions:** Mandibular involvement of neuroblastoma is rare but clinically important. In infants and young children, persistent or atypical (para-/peri)mandibular swelling should not be assumed to be odontogenic or inflammatory. Early imaging, biopsy, and interdisciplinary referral are essential for timely diagnosis and treatment.

## 1. Introduction

Neuroblastoma (NB) is one of the most common extracranial solid malignancies in early childhood, representing an embryonal tumor of the sympathetic nervous system derived from neural crest cells. It can arise at any point along the sympathetic nervous system, most frequently in the adrenal medulla or along the paravertebral sympathetic chain [1]. In Germany, an average of approximately 120 children were diagnosed with NB annually between 1980 and 2021, according to the German Childhood Cancer Registry [2]. The clinical course is highly variable, ranging from spontaneous regression or maturation—particularly in infants—to aggressive metastatic disease [3]. Using deep whole-genome sequencing, molecular clock analysis, and population–genetic modeling in a cohort of 100 patients, Körber et al. demonstrated that neuroblastomas across the entire clinical spectrum acquire aneuploidy as early as the first trimester of pregnancy, when the adrenal medulla forms from sympathetic neuroblasts [4].

At the time of diagnosis, a substantial proportion of patients already present with metastatic disease. Common metastatic sites include the bone, bone marrow, lymph nodes, liver, and skin, with skeletal and bone marrow involvement being particularly relevant in advanced-stage cases [5]. A characteristic biochemical feature of NB is the production and urinary excretion of catecholamine metabolites, specifically homovanillic acid and vanillylmandelic acid, which support the diagnostic work-up [6].

Manifestations of NB in the head and neck region are rare overall and may appear either as primary disease or, more frequently, as metastases [7,8,9]. Published cases describe mandibular swelling as the first sign of disseminated disease, and mandibular metastases have been reported to mimic odontogenic, inflammatory, or other oral and maxillofacial conditions [7]. Clinically, mandibular involvement may present with nonspecific findings such as facial or mandibular swelling, pain, tooth mobility, facial asymmetry, or radiographic bone destruction [7,8,10]. These symptoms can initially suggest odontogenic infection, parotitis, osteomyelitis, or eruption-related pathology, which may dangerously delay the diagnosis of the underlying malignancy.

For pediatric dentists, oral and maxillofacial surgeons, and other clinicians evaluating facial swelling in infants, awareness of this rare presentation is crucial. Progressive, atypical, or treatment-resistant mandibular swelling should prompt immediate imaging, biopsy when indicated, and interdisciplinary referral, especially given that most reported mandibular lesions represent metastatic disease rather than a primary tumor [7]. In this article, we present the case of an infant with high-risk adrenal NB initially presenting with a left paramandibular mass suspicious for parotitis. Additionally, we aimed to perform a systematic literature search and provide a narrative review of published pediatric cases of mandibular involvement in neuroblastoma, focusing on clinical presentation, diagnostic findings, treatment, and outcomes.

## 2. Case Report

### 2.1. Case Description

We present the case of an eight-month-old Caucasian girl diagnosed with metastatic neuroblastoma involving the mandible. She was born after an uneventful pregnancy at 42 weeks of gestation. Delivery was complicated by prolonged labor and ultimately required cesarean section. Birth weight was 4232 g and birth length 53 cm. Prior to presentation, the patient had no history of severe systemic disease, surgery, or hospitalization. The family history was negative for malignant disease. The patient had no siblings; her father was reported to be healthy, while her mother had a history of ulcerative colitis and chronic gastritis.

**Figure 1:**
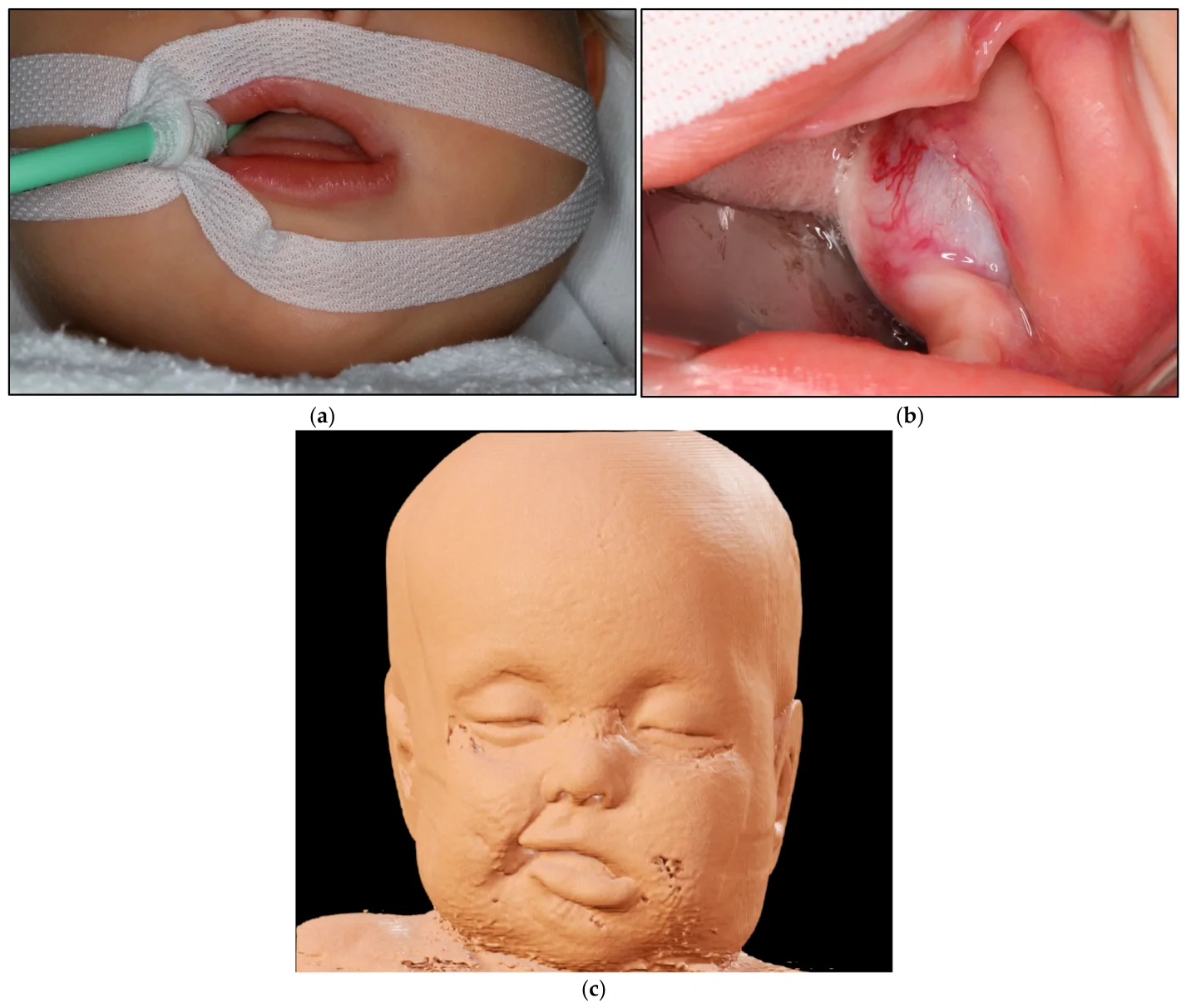
(**a**,**b**). Buccal swelling and mandibular mass on the left side. (**a**) Presentation with suspected parotitis; painless swelling of the cheek soft tissues. (**b**) Firm swelling of the edentulous mandible with a pearlescent surface, neovascularization, and indistinct borders. (**c**) Three-dimensional MRI reconstruction demonstrating the left-sided facial asymmetry associated with the mandibular lesion. Reconstruction generated using Brainlab^®^ Software Elements 7.0 (Brainlab AG, Munich, Germany).

In August 2019, the patient was transferred from a regional hospital to our institution after one week of unsuccessful antibiotic treatment for presumed parotitis. Despite antimicrobial therapy, the left-sided paramandibular swelling continued to increase in size. Clinical examination revealed a painless extraoral swelling of the left cheek (Figure 1a). Intraorally, a swelling of the still toothless posterior alveolar ridge with marked superficial vascularization was noted (Figure 1b). In addition to progressive facial swelling, the patient was in good general condition, afebrile, and showed no pain, nausea, vomiting, feeding difficulties, or other systemic symptoms. No cervical lymphadenopathy was detected. At presentation, body weight was 8.9 kg and body length was 70 cm. Cross-sectional imaging was performed because of the unusual clinical appearance.

**Figure 2:**
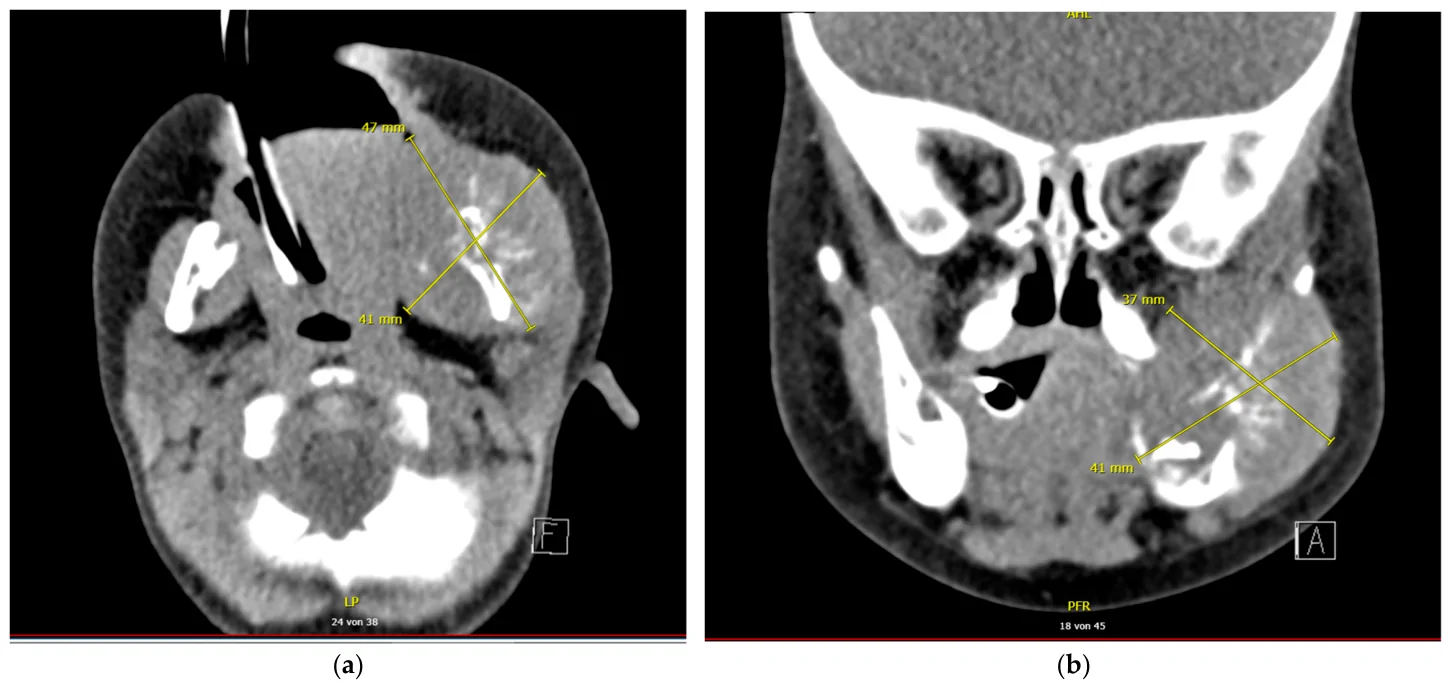

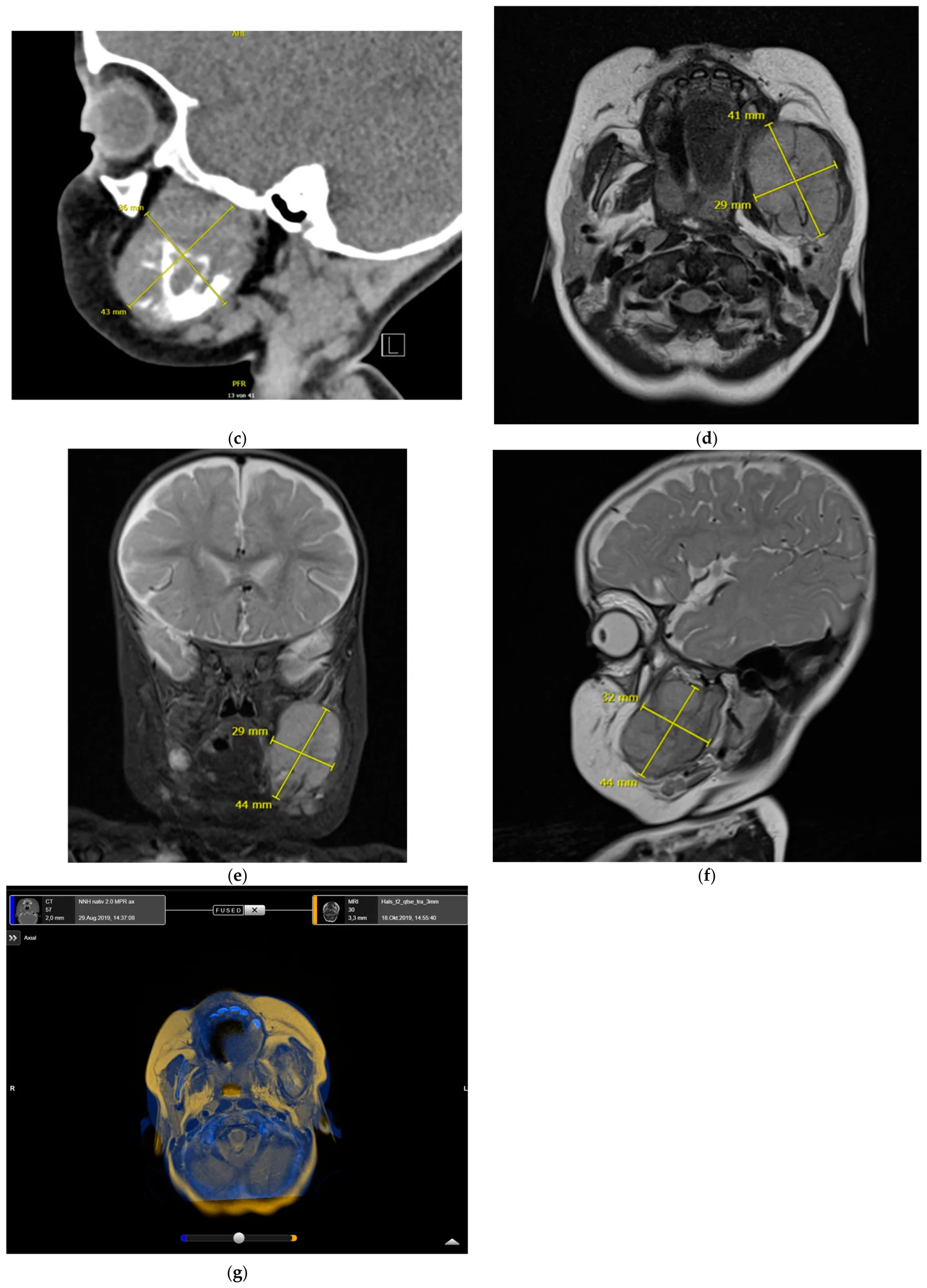
(**a**–**d**) CT (soft tissue window) and MRI. (**a**–**c**) CT: Mass measuring 47 × 30 × 41 mm in the left mandible with osteodestruction (“sunburst” periosteal reaction) and infiltration of the surrounding soft tissues. (**d**–**f**) MRI: Massive intraosseous mass of the left mandible with infiltration and displacement of the surrounding soft tissues. (**d**) Axial T2-weighted turbo spin-echo (TSE) sequence; (**e**) coronal T1-weighted turbo spin-echo (TSE) sequence; (**f**) sagittal T2-weighted turbo spin-echo (TSE) sequence. (**g**) Fused CT and MRI image; the CT dataset is displayed in blue and the MRI dataset in orange, illustrating the relationship between the osteodestructive mandibular lesion and the surrounding soft tissues. Image fusion was performed using the Brainlab^®^ Software Elements 7.0, Image fusion 5.0 (Brainlab AG, Munich, Germany). (University Clinic and Polyclinic for Diagnostic Radiology, University Hospital Halle; Dr. C. Kunze).

**Figure 3:**
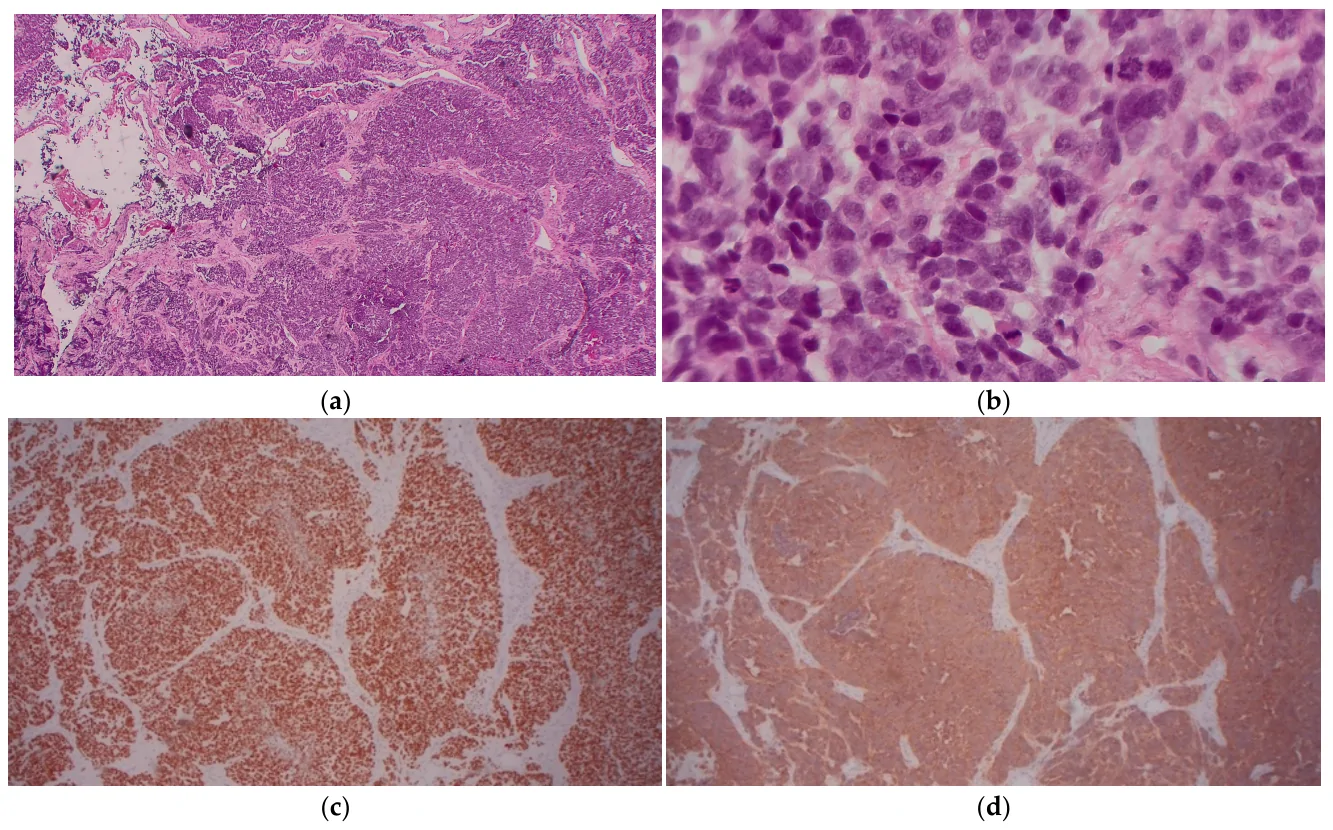
(**a**–**d**) Histopathological findings—undifferentiated neuroblastoma (according to INPC). (**a**,**b**) H&E staining: Fibrous tissue with nodular, lobularly arranged infiltrates of a small, round, blue-cell atypical population (25× and 400×, respectively). (**c**,**d**) Immunohistochemistry: strong nuclear expression of PHOX2B (50×) and cytoplasmic expression of synaptophysin (50×). (Institute of Pathology, Martin Luther University Halle-Wittenberg; Dr. C. Busse).

Contrast-enhanced CT and MRI demonstrated a mass measuring 47 × 30 × 41 mm involving the left mandibular angle with cortical bone destruction and sunburst periosteal reaction (Figure 2a–e). Based on the clinical and radiological findings, a malignant mesenchymal tumor, particularly a sarcoma, was initially considered in the differential diagnosis. An incisional biopsy was performed, and histological examination revealed the diagnosis of undifferentiated neuroblastoma (Figure 3a–d).

**Figure 4:**
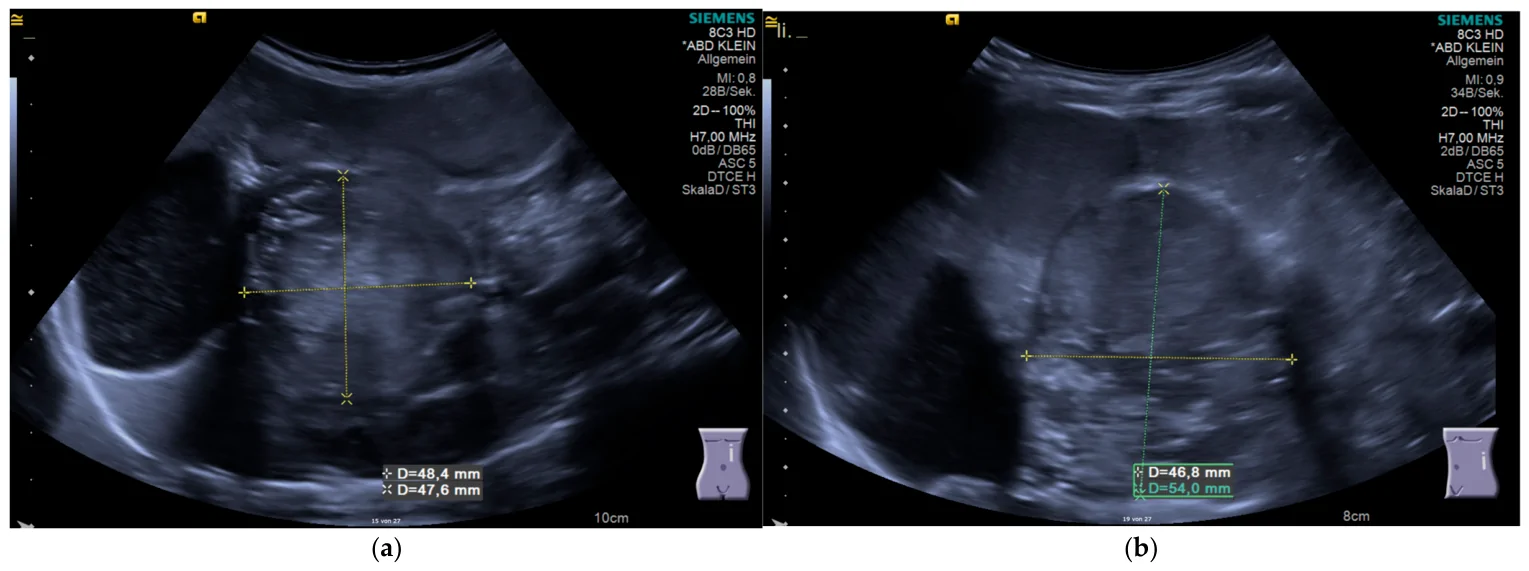
(**a**,**b**) Renal ultrasound. (**a**,**b**) Visualization of a mass measuring 48 × 47 × 54 mm in the left adrenal gland in two different longitudinal sections of the retroperitoneal space. Imaging was performed using an ACUSON S2000 ultrasound system (Siemens Healthineers, Erlangen, Germany). (University Clinic and Polyclinic for Diagnostic Radiology, Pediatric Radiology, University Hospital Halle; Dr. C. Kunze).

**Figure 5:**
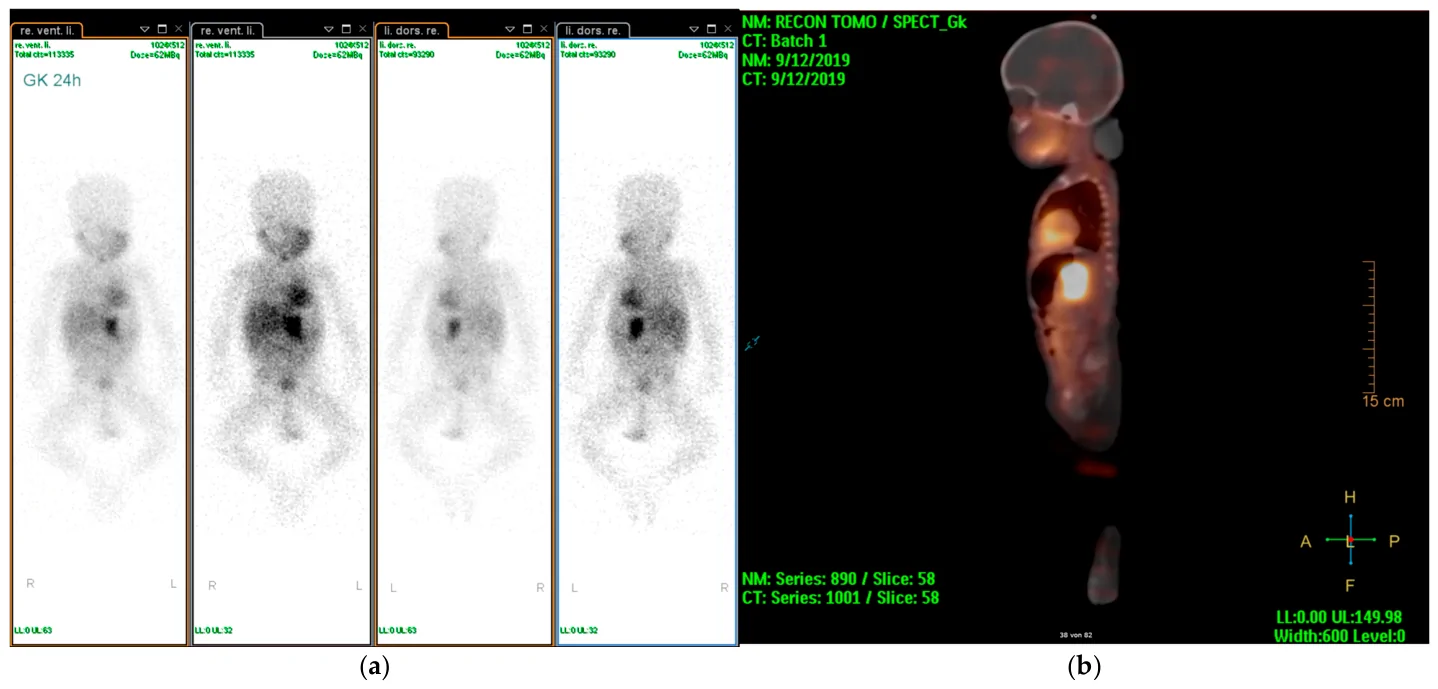
(**a**,**b**) Nuclear medicine findings. (**a**) ^123^I-metaiodobenzylguanidine (MIBG) scintigraphy: tracer uptake in the mandible (anterior and posterior views). (**b**) SPECT/CT: pathological tracer uptake in the mandible and the left adrenal gland. (Department of Radiation Medicine, Nuclear Medicine, University Hospital Halle; Dr. Ch. Geibig).

To determine the extent of disease, staging investigations were performed. Abdominal ultrasound demonstrated a left adrenal mass, which was identified as the primary tumor site (Figure 4a,b). This finding was supported by SPECT/CT imaging, which showed corresponding pathological tracer uptake in the left adrenal gland and the mandibular lesion (Figure 5b). Cytomorphological examination demonstrated extensive bone marrow infiltration.

Biochemical evaluation revealed markedly elevated urinary catecholamine metabolites: homovanillic acid (HVA) (16.3-fold), vanillylmandelic acid (VMA) (1.7-fold), and normetanephrine (6.5-fold). Molecular analysis by means of fluorescence in situ hybridization (FISH) demonstrated amplification of the prognostically relevant MYCN oncogene. Based on the presence of distant metastatic disease and MYCN amplification, the patient was classified as having high-risk stage IV neuroblastoma according to the International Neuroblastoma Staging System (INSS) [11].

### 2.2. Therapy and Clinical Course

The patient received multimodal treatment according to the NB2016 high-risk neuroblastoma protocol. Induction therapy included high-dose chemotherapy with busulfan and melphalan, followed by autologous stem cell transplantation and surgical resection of the left adrenal primary tumor from November 2019 to February 2020.

**Figure 6:**
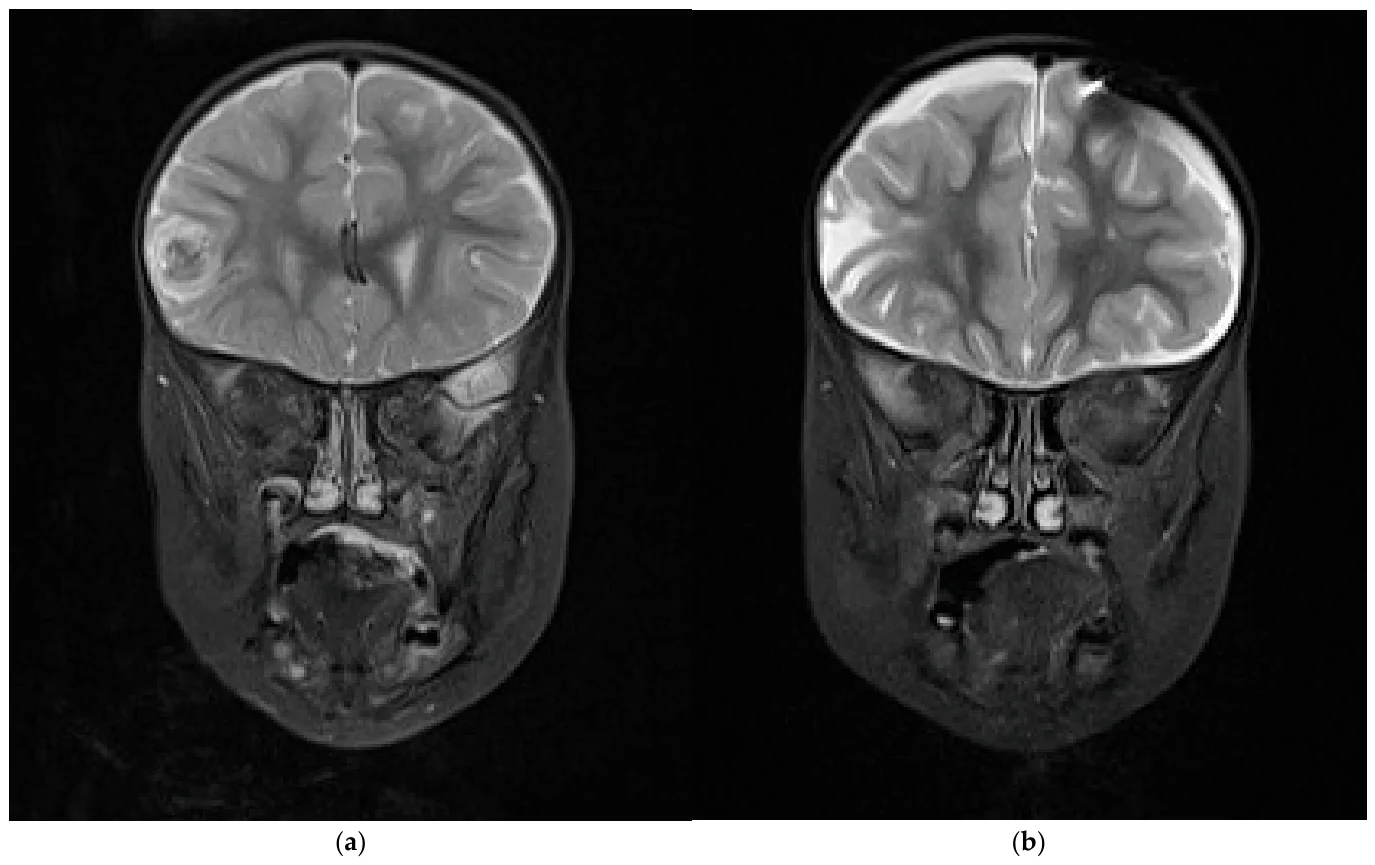
(**a**,**b**) Follow-up MRI findings of the cerebral metastasis. (**a**) Coronal T1-weighted magnetization-prepared rapid gradient-echo (MPRAGE) sequence showing a hemorrhagic metastasis in the right frontal region. (**b**) Coronal short tau inversion recovery (STIR) sequence demonstrating an unremarkable resection cavity in the right frontal region after surgical treatment. (University Clinic and Polyclinic for Diagnostic Radiology, University Hospital Halle.; Dr. C. Kunze).

In May and June 2020, the patient underwent percutaneous proton radiotherapy (30.6 Gy) to the submental region, the left mandibular metastasis, and the primary tumor region at the University Hospital Essen (Germany). Following radiotherapy, MRI demonstrated no pathological contrast enhancement of the mandibular lesion. However, a newly developed hemorrhagic metastasis in the right frontal lobe was subsequently detected and surgically resected in July 2020 (Figure 6a). The patient then received three cycles of irinotecan and temozolomide, followed by intraventricular radioimmunotherapy with ^131^I-omburtamab in January 2021. Additional treatment included five cycles of anti-GD2 immunotherapy with naxitamab, five cycles of maintenance therapy with temozolomide, and treatment with the ALK inhibitor lorlatinib, which required temporary interruptions because of progressive respiratory complications.

Follow-up MRI performed in November 2022 demonstrated an unremarkable right frontal resection cavity (Figure 6b) and persistent mild sclerosis of the left mandibular angle without evidence of progressive local disease. Whole-body ^123^I-MIBG scintigraphy including SPECT/CT showed physiological tracer distribution without evidence of MIBG-positive tumor manifestations. A whole-body MRI performed in August 2023 likewise demonstrated no evidence of recurrent neuroblastoma.

Following recurrent respiratory infections and progressive respiratory deterioration, interstitial pulmonary fibrosis was diagnosed in March 2023. Despite sustained radiological remission and the absence of recurrent neuroblastoma on follow-up imaging, the patient’s pulmonary fibrosis progressed, resulting in chronic respiratory insufficiency and the need for tracheostomy in July 2023. During the subsequent course, she required repeated hospitalizations because of progressive respiratory deterioration and became dependent on high-flow oxygen therapy. Lung transplantation was considered but was ruled out as a therapeutic option because of the recent recurrence of high-risk neuroblastoma with cerebral metastasis. She died shortly before the age of five years from complications of progressive interstitial pulmonary fibrosis.

A chronological overview of the patient’s diagnostic work-up, treatment, and clinical course is provided in Table 1.

## 3. Materials and Methods

### 3.1. Study Design

**Figure 7:**
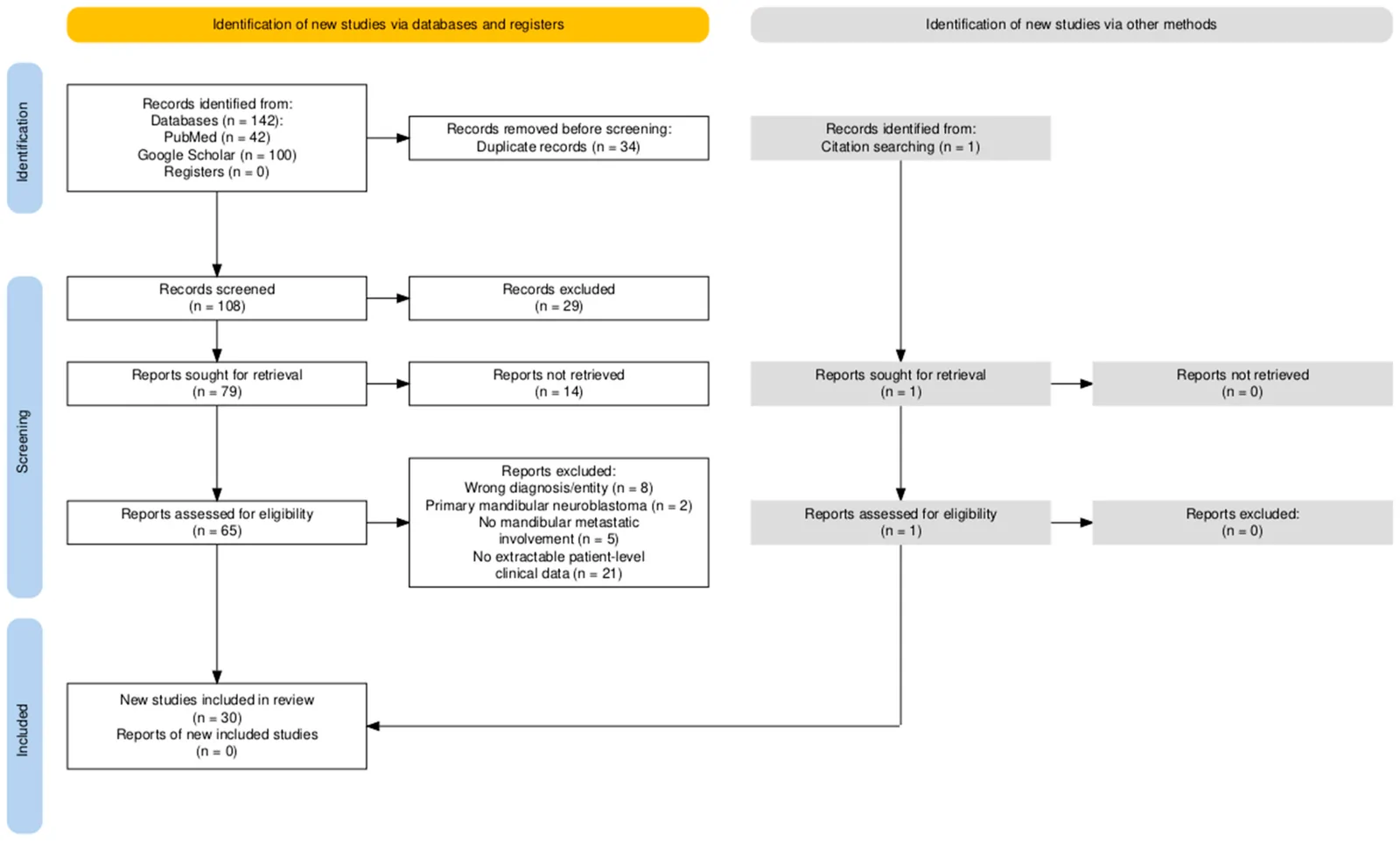
PRISMA 2020 flow diagram illustrating the literature search and study selection process for the narrative review. Adapted from Page et al. (2021) [12].

In this work, we combine a retrospective single-case report with a narrative literature review employing a systematic search strategy. The case report component follows established guidelines for anonymized clinical case reporting. The literature review component was not designed or registered as a systematic review or meta-analysis; given the rarity of the condition and the exclusively case-report-based evidence base, a narrative synthesis was considered the most appropriate approach. Nevertheless, a structured, reproducible search strategy and the PRISMA 2020 flow diagram were adopted to enhance transparency of study identification and selection (Figure 7).

### 3.2. Case Study

Clinical, radiological, histopathological, molecular, therapeutic, and follow-up data of the presented patient were retrieved retrospectively from their medical records. The case was prepared in accordance with the principles of anonymized clinical reporting. The case description was prepared in accordance with the CARE guidelines, covering patient information, clinical findings, timeline, diagnostic assessment, therapeutic interventions, follow-up, and outcomes [13]. Patient-specific information was de-identified before manuscript preparation.

### 3.3. Literature Search

The systematic literature search was initiated in February 2026 and completed on 16 May 2026. The literature search was conducted in PubMed and Google Scholar. The research question and eligibility criteria were structured according to the PICOST framework. As the objective of this focused review was to summarize published pediatric cases of mandibular metastases from neuroblastoma, no comparison group was applicable.

The PICOST components were defined as follows: Population: pediatric patients (age 0–17 years) diagnosed with neuroblastoma. Intervention/Exposure: mandibular metastatic involvement. Outcome: clinical presentation, diagnostic work-up, treatment, and clinical outcome. Study design: case reports and case series. Time: no restriction on publication date; all eligible studies published up to the final search date were considered for inclusion.

PubMed served as the primary database for this review. For the PubMed search, Medical Subject Headings (MeSH) and free-text terms were combined using Boolean operators (AND/OR). The search strategy included terms related to neuroblastoma, mandibular involvement (mandible, mandibular, jaw), and the pediatric population (child, infant, adolescent). The following search string was applied on 16 May 2026:

(“Neuroblastoma”[MeSH] OR neuroblastoma[Title/Abstract]) AND (“Mandible”[MeSH] OR mandible[Title/Abstract] OR mandibular[Title/Abstract] OR jaw[Title/Abstract]) AND (“Child”[MeSH] OR “Infant”[MeSH] OR “Adolescent”[MeSH] OR child[Title/Abstract] OR pediatric[Title/Abstract] OR paediatric[Title/Abstract] OR infant[Title/Abstract] OR adolescent[Title/Abstract]).

This search retrieved 42 records. No language or date filters were applied at the database level; language-based exclusion was performed subsequently during the screening phase (see Section 3.4).

Google Scholar was used as a complementary search engine to identify additional studies not indexed in PubMed, including older or less widely indexed case reports, using the search term “neuroblastoma mandible”. Given the broader and less structured nature of Google Scholar search results, screening was limited to the first 100 results, sorted by relevance.

Duplicate records (*n* = 34) were removed prior to screening. Additionally, the reference lists of eligible publications were manually screened to identify further relevant reports not retrieved by the database search.

### 3.4. Eligibility Criteria

Publications were considered eligible for the tabular analysis if they described pediatric patients (age 0–17 years) with neuroblastoma metastasis involving the mandible. Cases were included if mandibular involvement was documented clinically, radiologically, histopathologically, or by means of nuclear medicine imaging.

Reports were excluded if they (1) described patients aged 18 years or older, (2) did not report mandibular metastatic involvement, including lesions confined to other head and neck sites without mandibular disease, (3) did not provide sufficient evidence to confirm the diagnosis of metastatic neuroblastoma involving the mandible, (4) did not report sufficient individual patient-level clinical information to allow extraction of the predefined study variables, or (5) were unavailable in English and did not provide an English abstract containing sufficient information for eligibility assessment and data extraction.

Articles published in languages other than English were not excluded if the English abstract contained sufficient information to confirm mandibular metastatic neuroblastoma and allowed extraction of the predefined study variables (see Section 3.5). No additional data were extracted from full-text articles that were not available in English in order to avoid potential inaccuracies resulting from translation. The potential risk of language bias associated with this approach is acknowledged as a limitation of the review.

Reports describing primary mandibular neuroblastoma or other neuroblastic entities, including ganglioneuroblastoma and ganglioneuroma, were excluded from the comparative analysis but are discussed separately because of their diagnostic and biological relevance.

### 3.5. Data Extraction

For each eligible case, the following variables were extracted when available: year of publication, country of origin, authors, patient age and sex, mandibular laterality, timing of mandibular metastasis in relation to the initial diagnosis, clinical presentation, site of the primary tumor, treatment, and outcome. Mandibular timing was categorized as involvement at initial diagnosis, occurring later during the disease course, or at recurrence/follow-up. Laterality was recorded as left, right, or bilateral, based on the mandibular side(s) reported to be involved. For lesions predominantly unilateral but with radiographic extension across the midline, laterality was recorded according to the predominant or initially described side; cases with genuine bilateral mandibular involvement were recorded as bilateral.

Treatment was summarized using broad categories, including chemotherapy, radiotherapy, surgery, autologous stem cell transplantation, immunotherapy, palliative chemotherapy, or no treatment when management was declined. If specific information was unavailable, the variable was recorded as “not reported”.

The literature search and data extraction were performed by a single/first author (R.M.). Therefore, disagreements between authors did not arise. Methodological questions and data interpretation were discussed with the senior authors during manuscript preparation. Although the retrospective cohort study by Singh et al. [9] represents one of the largest published series of pediatric mandibular metastatic neuroblastoma, it was not incorporated into the comparative case table because the publication provides only aggregated cohort data without extractable individual patient characteristics. The study was therefore considered narratively where appropriate throughout the manuscript.

### 3.6. Data Synthesis

Due to the fact that most of the available literature comprised isolated case reports and small case series with heterogeneous reporting, the data were summarized in a descriptive manner. Special emphasis was placed on identifying recurring clinical patterns, treatment methods, and reported outcomes. The review was reported in accordance with the PRISMA 2020 statement where applicable. Given that all included studies were case reports or small case series, a formal risk-of-bias assessment using standardized instruments was not performed, as such tools are not applicable to this study type; this factor is acknowledged as a limitation.

## 4. Results

Through our literature search, we identified 30 publications describing 31 pediatric cases of mandibular metastatic neuroblastoma, published between 1961 and 2024. In addition, reports describing primary mandibular neuroblastoma and other neuroblastic entities, including ganglioneuroblastoma and ganglioneuroma, were identified. These special cases were not included in the main comparative table but are addressed separately in the discussion. The present patient represents an additional, previously unpublished case and is therefore described separately in the Case Presentation section. The included cases originated from multiple countries and were published over more than six decades, reflecting the rarity of mandibular metastatic neuroblastoma and the limited evidence available in the literature.

The age of the reported patients ranged from 8 months to 15 years, with most cases occurring in infants and young children. The median age was approximately 3 years. Sex was reported in almost all cases, with 20 male and 10 female patients; in one case, sex was not reported. Mandibular involvement was reported more frequently on the left side than on the right, with left-sided involvement in 18 cases, right-sided involvement in 10 cases, and bilateral involvement in 3 cases.

In most cases, mandibular involvement was already present at the time of initial diagnosis. Overall, 26 cases were classified as mandibular metastasis at initial diagnosis, including one clinically occult lesion identified only post mortem, whereas 5 cases developed mandibular involvement later during the disease course or at recurrence. The primary tumor was most commonly located in the adrenal gland when reported, with a similar distribution between left- and right-sided adrenal primaries. Only one patient was reported to have a primary tumor in the periorbital region. In a small number of cases, the primary site was not reported, not further specified, or could not be clearly determined.

As all cases included in the main analysis involved mandibular metastatic disease, they corresponded to disseminated neuroblastoma and would generally be classified as advanced-stage disease, corresponding to stage 4 according to the INSS when staged using this system.

The clinical presentation was variable but demonstrated recurring oral and maxillofacial manifestations. The most common findings included mandibular or facial swelling, intraoral or gingival masses, pain, tooth mobility or displacement, and symptoms mimicking odontogenic or inflammatory disease. In several cases, initial suspicion was for dental abscess, odontogenic infection, or Bell’s palsy, and a lack of response to antibiotic treatment was commonly noted. Systemic manifestations included fever, malaise, weight loss, anemia, generalized bone pain, neurological signs, or abdominal complaints.

Treatment strategies reflected the evolution of neuroblastoma management over more than six decades and included chemotherapy, radiotherapy, adrenalectomy or resection of the primary tumor, autologous stem cell transplantation, MIBG therapy, immunotherapy, mandibular surgery, palliative chemotherapy, or no treatment when further management was declined. Outcomes were heterogeneous and frequently incompletely reported. Among the included cases, 15 patients died, often within weeks or months after diagnosis or treatment initiation, whereas 7 cases reported remission, no evidence of disease, tumor-free follow-up, or clinical improvement. In nine cases, the final outcome was not reported or remained unclear.

A condensed overview of the included metastatic cases is provided in Table 2; a detailed version including all extracted variables for each case is available in Table S1 (Supplementary Materials).

## 5. Discussion

The reviewed literature demonstrates a recurring clinical pattern: mandibular metastasis from neuroblastoma frequently presents with nonspecific oral and maxillofacial findings and is therefore initially mistaken for odontogenic or inflammatory disease. The clinical presentation of the present patient closely reflected this pattern. In the reviewed cases, mandibular or facial swelling, intraoral masses, tooth mobility or displacement, and pain were among the most frequently described oral and maxillofacial findings. Multiple cases were initially interpreted as dental abscesses, odontogenic infections, Bell’s palsy, or other benign conditions. In addition, a lack of response to antibiotic treatment was repeatedly reported. This diagnostic challenge was also reflected in the reported cases summarized in Table 2. Beyond these recurring oral findings, individual cases also exhibited more specific extraoral or systemic signs suggestive of disseminated neuroblastoma, including periorbital ecchymosis, proptosis, scalp swelling, neurological symptoms, or generalized bone pain. Periorbital ecchymosis, sometimes referred to as “raccoon eyes,” may occur in neuroblastoma due to orbital or periorbital metastatic infiltration with associated hemorrhage and is therefore an important clinical warning sign of disseminated disease [41].

Mandibular metastasis was most often already present at initial diagnosis rather than occurring exclusively as a late relapse. Specifically, mandibular involvement was documented at initial diagnosis in 26 of the 31 reviewed cases, providing strong evidence that oral or maxillofacial symptoms may represent one of the earliest clinically apparent signs of disseminated neuroblastoma. Most reported primary tumors were located in the adrenal gland or abdomen, consistent with the typical abdominal origin of neuroblastoma [5].

The differential diagnosis of mandibular or facial swelling in young children is broad and includes both benign inflammatory conditions and malignant disease. Odontogenic infection, osteomyelitis, eruption-related pathology, salivary gland disease, and benign odontogenic or non-odontogenic jaw lesions are often considered initially. However, malignant entities should be considered, particularly when swelling is rapidly progressive, atypical, associated with systemic symptoms, or unresponsive to antibiotics. In the head and neck region, Burkitt lymphoma is an important pediatric differential diagnosis, as it may present with rapidly enlarging jaw or facial masses [42]. In children with an abdominal or retroperitoneal mass, nephroblastoma/Wilms tumor should also be considered [43]. Although mandibular metastasis is not a typical presenting feature, reported cases exist [44]. Other relevant malignant differential diagnoses include leukemia, lymphoma, Ewing sarcoma, osteosarcoma, rhabdomyosarcoma, and Langerhans cell histiocytosis [45,46,47,48,49]. Therefore, biopsy and immunohistochemical evaluation remain essential when clinical or radiological findings are atypical or progressive.

A further diagnostic consideration is distinguishing between primary and metastatic neuroblastoma of the mandible. This distinction is important, as it influences staging, treatment approach, and prognosis. True primary lesions in the mandible represent localized disease, while mandibular metastases are a sign of widespread neuroblastoma [11]. Most reported cases in the literature represent metastases from adrenal primary tumors. In rare situations, cases are classified as primary mandibular neuroblastoma; however, confirming this diagnosis is challenging. Particularly in older reports with limited staging information, occult primary tumors or early metastasis may not have been excluded by the diagnostic techniques available at that time. Therefore, cases labeled as primary mandibular neuroblastoma were excluded from the main comparative table of metastatic cases, though they remain relevant as part of the spectrum of neuroblastic tumors in the mandible.

The biological mechanisms underlying mandibular metastasis in neuroblastoma remain incompletely understood. In general, metastatic dissemination is a multistep process involving tumor cell detachment from the primary site, local invasion, intravasation, survival within the circulation, extravasation, and colonization of distant tissues [50]. More recent research suggests that organ-specific metastasis is additionally influenced by interactions between tumor cells and the target organ microenvironment. In particular, extracellular vesicles have emerged as important mediators of bone tropism and metastatic progression and may represent potential therapeutic targets [51]. Furthermore, recent studies indicate that distinct tumor cell clones and organ-specific signaling pathways contribute to metastatic heterogeneity and the preferential colonization of organs such as bone [52]. Consistent with these biological concepts, in a retrospective analysis of 1224 patients with neuroblastoma, bone was identified as one of the most common metastatic sites, although mandibular involvement was not analyzed separately [53]. These findings may partly explain why the mandible, as part of the skeletal system, can represent a site of metastatic involvement in disseminated neuroblastoma.

The biological behavior and clinical course of neuroblastoma are highly heterogeneous, ranging from spontaneous regression or maturation to aggressive metastatic progression [6]. The present patient was staged according to the INSS, the system applied at the time of diagnosis and underlying most of the historical cases reviewed herein. Contemporary practice additionally employs the International Neuroblastoma Risk Group Staging System (INRGSS), a pretreatment, imaging-based system that defines stages according to image-defined risk factors rather than surgical outcome [54]. Accordingly, current risk stratification integrates both clinical and biological factors, including disease stage, age at diagnosis, tumor histopathology, MYCN status, DNA index, and tumor ploidy, to guide treatment intensity and prognostic assessment [55]. Among the biological risk factors incorporated into the revised Children’s Oncology Group risk classification system, amplification of the MYCN proto-oncogene is associated with aggressive disease behavior and represents a key marker for risk stratification. Further high-risk features include advanced disease stage, age older than 18 months at diagnosis, distant metastases, and additional molecular or chromosomal aberrations, such as 1p deletion [56]. In the present patient, MYCN amplification was detected, consistent with classification as high-risk neuroblastoma.

In some patients, neuroblastoma may undergo spontaneous maturation or treatment-induced differentiation into a more mature, less aggressive histological phenotype [56]. Therefore, a mandibular lesion with ganglioneuromatous histology does not necessarily represent a primary benign mandibular tumor. Instead, it may reflect maturation of a previous metastatic neuroblastoma lesion, particularly when disseminated disease had been documented at initial diagnosis [57]. Such cases illustrate the biological plasticity of neuroblastic tumors but are not directly comparable with conventional metastatic mandibular neuroblastoma. Likewise, reports describing ganglioneuroblastoma or ganglioneuroma represent biologically distinct entities with different degrees of cellular maturation and were therefore not included in the main tabular analysis. Nevertheless, these lesions remain relevant for the differential diagnosis of mandibular neuroblastic lesions.

Therapeutic strategies for neuroblastoma vary according to risk group and have evolved considerably over recent decades. Treatment may range from observation to intensive multimodal therapy, including chemotherapy, surgery, radiotherapy, autologous stem cell transplantation, and immunotherapy [58]. In high-risk neuroblastoma, anti-GD2 immunotherapy has become an important component of consolidation therapy after autologous stem cell transplantation and aims to reduce minimal residual disease and relapse risk [59,60]. In addition to conventional multimodal treatment, molecularly targeted therapies, including ALK inhibitors, have emerged as promising treatment options for selected patients with high-risk neuroblastoma [61]. This development is reflected in the present case, in which the patient received the ALK inhibitor lorlatinib as part of the treatment course. Nevertheless, intensive multimodal therapy is associated with substantial acute and long-term toxicity [62,63]. In the present patient, progressive interstitial pulmonary fibrosis represented a severe therapy-associated complication and illustrates the potential morbidity of high-risk neuroblastoma treatment.

Historically, jaw or mandibular metastases of neuroblastoma were often associated with an unfavorable prognosis. However, this interpretation was largely based on individual case reports and small case series from treatment eras before modern risk-adapted multimodal therapy [8,9]. Given the rarity of mandibular metastatic neuroblastoma and the limited number of published cases, the independent prognostic significance of mandibular involvement remains difficult to determine. Staging systems, imaging modalities, molecular diagnostics, treatment protocols, and follow-up periods differed substantially across the available reports, precluding direct comparison of outcomes. In the present review, outcomes were heterogeneous, ranging from complete remission or no evidence of disease during follow-up to death within weeks or months after diagnosis or treatment initiation. Notably, in earlier reports, many of which were published before the introduction of contemporary risk-adapted treatment protocols, death within weeks or months after diagnosis is frequently described. More recent publications demonstrate that prolonged survival and complete remission are achievable with multimodal treatment in selected patients. However, fatal outcomes continue to occur despite modern therapeutic approaches. Taken together, these observations suggest that prognosis is more likely determined by systemic disease burden, tumor biology, age, treatment response, and treatment-related complications than by mandibular localization alone, despite substantial advances in diagnostic evaluation, molecular risk stratification, and therapy over the past six decades [9]. The present patient was classified as high-risk because of metastatic disease and MYCN amplification. Despite major advances in multimodal therapy, patients in this risk group, irrespective of mandibular involvement, continue to have a reported 5-year overall survival of approximately 63% according to the revised Children’s Oncology Group risk classification system [56].

From an oral and maxillofacial perspective, mandibular metastases may cause specific local morbidity, including tooth mobility, tooth displacement, disturbed dentition, intraoral masses, facial asymmetry, pain, destructive mandibular lesions, and, in extensive cases, impairment of oral function, aesthetics, dental development, or airway safety. Long-term survivors may therefore require dental, orthodontic, prosthetic, or reconstructive rehabilitation, depending on the extent of mandibular involvement and the local effects of surgery, radiotherapy, or tumor destruction [9]. In addition, survivors of high-risk neuroblastoma require surveillance for late effects, including pulmonary complications, endocrinopathies, hearing loss associated with platinum-based chemotherapy, growth and developmental disturbances, dental abnormalities, and secondary malignancies [64,65]. The clinical course of the present patient demonstrates that even when radiological remission is achieved, treatment-associated morbidity may remain severe. This finding is in line with the recent narrative review by De Bona et al. (2026), who emphasize that despite advances in multimodal therapy, high-risk and relapsed neuroblastoma remain associated with substantial morbidity and mortality [66].

To our knowledge, this review includes the largest collection of individually reported pediatric cases of mandibular metastatic neuroblastoma summarized to date, enabling the identification of recurring clinical patterns despite the rarity of this condition.

Despite its strengths, this review has several limitations. The available literature consists almost exclusively of case reports and small case series, many of which are historical and incompletely reported. Clinical presentation, imaging findings, treatment, and outcome were inconsistently described. Excluding these reports would have substantially reduced the number of available cases, however, given the rarity of mandibular metastatic neuroblastoma in children. We included only reports available in English or with an English abstract that provided sufficient information for eligibility assessment and data extraction. The potential risk of language bias associated with this approach is acknowledged as a limitation of this narrative review. Because the included cases were derived from heterogeneous case reports with variable reporting completeness, missing data may have introduced reporting bias and limited direct comparability across cases. In addition, diagnostic imaging, staging systems, molecular risk stratification, and treatment protocols have changed over time. Therefore, no conclusions regarding treatment efficacy or the independent prognostic significance of mandibular involvement can be drawn from the available data. Furthermore, the Google Scholar search was pragmatically limited to the first 100 results, sorted by relevance; although this is a common approach given the platform’s lack of structured indexing, it cannot be excluded that additional relevant records exist beyond this threshold. Finally, study selection and data extraction were conducted by a single author, potentially increasing the risk of reviewer-related bias despite the use of predefined eligibility criteria.

Despite these limitations, the present case and the reviewed literature emphasize that mandibular swelling, tooth mobility, pain, intraoral masses, or destructive jaw lesions in infants and young children should not exclusively be attributed to odontogenic or inflammatory disease. Although benign and inflammatory causes are far more common, atypical, progressive, systemic, or therapy-resistant findings should prompt early imaging, biopsy, and interdisciplinary evaluation.

## 6. Conclusions

Mandibular metastasis is a rare but clinically important manifestation of pediatric neuroblastoma. It may present with nonspecific oral or maxillofacial symptoms, including mandibular swelling, intraoral masses, pain, tooth mobility, or tooth displacement, and may mimic odontogenic or inflammatory disease. Persistent, atypical, or rapidly progressive mandibular swelling in young children should therefore prompt early imaging, histopathological confirmation, and interdisciplinary referral.

The present case highlights the diagnostic relevance of oral and maxillofacial findings in high-risk neuroblastoma and illustrates the complexity of multimodal therapy and long-term morbidity. Although mandibular involvement is often associated with disseminated disease, the available literature does not allow conclusions regarding its independent prognostic significance.

## Figures and Tables

**Table 1:** Timeline of diagnosis, treatment, and clinical course.

Date	Key Clinical Event
August 2019	Progressive left-sided paramandibular swelling; initially treated as parotitis with antibiotics without clinical improvement. CT and MRI demonstrated an osteodestructive lesion of the left mandible. Incisional biopsy performed.
September 2019	Histological diagnosis of undifferentiated neuroblastoma; staging revealed left adrenal primary tumor, bone marrow involvement, and MYCN amplification; classified as INSS stage IV high-risk disease.
September 2019–February 2020	Induction chemotherapy (NB2016 high-risk protocol; busulfan/melphalan), followed by autologous stem cell transplantation and resection of the adrenal primary tumor.
May 2020–June 2020	Percutaneous proton radiotherapy (30.6 Gy) to the primary site, the submental region, and left mandibular metastasis.
July 2020	Cerebral metastasis diagnosed and surgically resected (Figure 6a).
August 2020	Three cycles of irinotecan/temozolomide.
January 2021	Intraventricular radioimmunotherapy with ^131^I-omburtamab.
2021	Anti-GD2 immunotherapy (naxitamab) followed by maintenance therapy with temozolomide. Lorlatinib therapy initiated (later intermittently interrupted because of pulmonary toxicity).
November 2022	MRI and MIBG SPECT/CT; no evidence of active disease.
March 2023	Following recurrent respiratory infections and progressive respiratory deterioration, interstitial pulmonary fibrosis was diagnosed.
July 2023	Tracheostomy because of progressive chronic respiratory insufficiency.
August 2023	Whole-body MRI showed no evidence of recurrent neuroblastoma.
November 2023	Death due to progressive treatment-associated interstitial pulmonary fibrosis despite radiological remission of neuroblastoma.

**Table 2:** Reported pediatric cases (*n* = 31) of metastatic neuroblastoma involving the mandible.

Study	Age/ Sex	Mand. Site	Timing	Clinical Presentation	Primary Tumor	Tx	Outcome
Pedler, 1961 [14]	2.5 y/M	R	Initial dx	Malaise; fatigue; irritability; abdominal pain; lower R gingival swelling; loose teeth.	R adrenal NB	n.r.	Dec. 25 d after dx; cause: anemia/wasting.
Snyder et al., 1975 [15]	7 y/M	L	Initial dx (clinically occult; identified post mortem)	Pallor; vomiting; abdominal pain; large L abdominal mass.	L adrenal NB	RT, CTx; resecting n.p.	Dec. 10 wk after admission; cause: disease progression.
Hardt, 1976 [16]	5 y/M	R	Initial dx	Extraoral R mandibular swelling; intraoral swelling of R gingiva.	L adrenal NB	CTx, RT	Dec. 8 mo after dx; cause: pulmonary edema/jaundice/ascites.
Reich et al., 1981 [17]	13 y/F	R	Recurrence, 6 y after dx	Tingling/paresthesia of R mandibular gingiva, teeth, and lower lip; discomfort; mobility of R mandibular 2nd premolar and 1st molar.	L adrenal NB	CTx, RT, ASCT	Dec. 6 y after dx; shortly after detection of mand. metastasis.
Boraz, 1985 [18]	2.5 y/M	bilat.	Initial dx	Fever; lethargy; anorexia; weight loss; lower-extremity weakness; enlarging L temporal mass; ulcerated gingival lesion in R mandibular molar region.	R adrenal NB	CTx, RT	Dec. 5 mo after dx; cause: cardiac failure due to intraoral tumor hemorrhage.
Newland et al., 1985 [19]	6 y/M	L	Initial dx	Intermittent fever; tachycardia; no response to AB.	L adrenal NB	CTx, RT	Initial good Tx response; recurrence after 1 y; dec. 1.5 y after dx; cause: tumor progression.
Campbell et al., 1987 [20]	2 y/F	L	Initial dx	Mand. swelling; intermittent fever; tachycardia; no response to AB.	L adrenal NB	CTx, ADX, ASCT	NED at 2 y FU.
Tatsumi et al., 1989 [21]	8 y/F	bilat.	Initial dx	Bilateral mobility of lower permanent teeth.	R abdominal tumor, n.s.	CTx	Dec. 2 mo after dx.
Borle et al., 1991 [22]	2.5 y/M	L	Initial dx	L mand. swelling; fever; weight loss; loss of appetite; displaced L mand. molar.	n.r.	RT	Dec. 3 wk after Tx start; cause: cachexia.
Chan et al., 1994 [23]	2 y/F	L	Initial dx	Mild–moderate pain; L mand. intraoral soft-tissue mass; mobility of L primary 1st/2nd molars; no response to AB.	Suprarenal lesions; primary site n.d.	CTx, RT, surgery n.p.	Remission at 18 mo FU; final outcome n.r.
Ogunsalu & Smith, 1999 [24]	21 mo/M	L	Recurrence, 4 mo after dx	Prognathic mandible; intraoral L anterior mand. swelling with buccal/lingual expansion; gross tooth displacement.	L adrenal NB	RT, CTx; debulking n.p.	Initial good Tx response; recurrence after 4 mo; dec. at 2 y 10 mo.
Pellegrino et al., 2000 [25]	6 y/F	L	Initial dx	L mand. swelling mimicking dental abscess; hard bony expansion; mobility of L mand. 1st molar.	L adrenal NB	ADX, CTx, RT, ASCT	NED at 30 mo FU.
van den Berg et al., 2007 [26]	9 mo/M	R	Initial dx	R mand. swelling; feeding difficulties; bilateral orbital hematomas; abdominal mass; scrotal mass.	L adrenal NB	MIBG Tx, ADX, CTx, ASCT	NED at 13 y FU.
Otmani & Khattab, 2007 [27]	3 y/M	L	Initial dx	Anemia; generalized bone pain; L mand. swelling with tooth displacement; cervical LAD.	L adrenal NB	Palliative CTx	Dec. 1 mo after Tx start.
Baber et al., 2008 [28]	15 y/F	L	Initial dx	Painful swelling of L face; no response to AB.	R adrenal NB	CTx; ASCT planned	n.r.
Parker et al., 2011 [29]	8 mo/M	R	Initial dx	5 wk history of R mand. swelling.	R adrenal NB	CTx, ADX	NED at 2 y FU.
Kürklü et al., 2011 [10]	15 mo/M	L	Initial dx	Tooth mobility of primary maxillary and mand. incisors and R mand. molar.	R adrenal NB	CTx	Dec. 3 mo after dx; cause: progressive disease.
Dwojak et al., 2012 [30]	3 y/M	bilat.	Initial dx	Facial nerve paresis diagnosed as Bell’s palsy; facial swelling; ear/jaw pain; R floor-of-mouth/buccal-space mass; cervical LAD.	n.r.	n.r.	n.r.
Manor et al., 2012 [8]	7 y/M	L	Initial dx	L mand. mass; displaced tooth 36.	L adrenal NB	CTx, ADX	n.r.
Piccardo et al., 2014 [31]	6 y/M	R	Recurrence	Imaging-detected relapse; no mand. symptoms reported.	n.r.	CTx, 131I-MIBG Tx	n.r.
Mittal et al., 2015 [32]	3 y/F	R	Initial dx	3 mo history of R mand. mass.	R adrenal NB	CTx	Dec. 6 mo after Tx start; cause: disease progression.
Wade et al., 2018 [33]	11 mo/F	R	Initial dx	Progressive R proptosis; scalp swelling.	L adrenal NB	CTx, ADX	Dec. 6 mo after Tx start.
Waldron et al., 2019 [34]	20 mo/F	L	Initial dx	R temporal mass mimicking cephalohematoma; esotropia; periorbital ecchymosis; gait changes; L mandibular lesion on MRI.	L adrenal NB	CTx, ADX, ASCT, RT, IT, retinoic acid	CR; 3 mo scan negative.
Msallem et al., 2020 [35]	34 mo/M	L	Initial dx	L cheek swelling; painful posterior vestibular mass; shift of primary L 2nd molar; no response to AB.	L adrenal NB	CTx, ASCT, RT, IT	NED 36 mo after dx.
do Amaral-Silva et al., 2021a [7]	4 y/M	L	Initial dx	2 wk history of painful swelling of L mandible; generalized body aches; weakness.	L adrenal NB	CTx, ADX, ASCT, RT	NED at 2 y FU.
do Amaral-Silva et al., 2021b [7]	3 y/M	L	Initial dx	1 mo history of L mand. swelling.	R adrenal NB	CTx	Significant improvement after Tx; final outcome n.r.
Ullah et al., 2021 [36]	3 y/M	L	Initial dx	L submand. swelling for 1 mo; back/leg pain; fever; urinary retention/constipation; no response to AB.	R adrenal NB	CTx, ADX, ASCT, RT, IT	Clinical improvement at last FU; final outcome n.r.
Sun et al., 2021 [37]	4 y/F	L	Recurrence, 9 mo after dx	After initial tumor-free interval: leg paresis; persistent maxillofacial pain; swelling of L mandible.	R adrenal NB	Initial CTx; recurrence treated with RT	Dec. 2 y after dx.
Simre et al., 2021 [38]	3 y/n.r.	L	Initial dx	Increasing tender mand. mass; fever; weight loss.	R adrenal NB	No Tx; further management refused	n.r.
Izi et al., 2023 [39]	3 y/M	R	Initial dx	Scalp swelling; fixed painful mand. mass.	R adrenal NB	n.r.	n.r.
de Castro-Rodrigues et al., 2024 [40]	14 mo/M	R	Later during disease course	Rapidly enlarging R mand. swelling; trismus, pain; impaired oral intake; tooth mobility.	Periorbital	CTx; palliative care after mand. met.	Dec. 8 wk after diagnosis of mand. met.; cause: airway compromise.

Abbreviations: AB, antibiotics; ADX, adrenalectomy; ASCT, autologous stem cell transplantation; CTx, chemotherapy; d, day(s); dec., deceased; dx, diagnosis; FU, follow-up; IT, immunotherapy; mand., mandibular; mo, month(s); NB, neuroblastoma; NED, no evidence of disease; n.d., not determined; n.p., not possible; n.r., not reported; n.s., not specified; RT, radiotherapy; Tx, treatment/therapy; wk, week(s); y, year(s). Note: Laterality was recorded as left, right, or bilateral, based on the mandibular side(s) reported to be involved. For lesions predominantly unilateral but with radiographic extension across the midline, laterality was recorded according to the predominant or initially described side; cases with genuine bilateral mandibular involvement were recorded as bilateral. Reports describing primary mandibular neuroblastoma, ganglioneuroblastoma, or ganglioneuroma were excluded from this comparative table but are discussed separately.

## Data Availability

The data supporting the literature review are derived from previously published case reports and are summarized in Table 2. Additional extracted data are available from the corresponding author upon reasonable request. Patient-specific clinical data from the present case are not publicly available due to privacy and ethical restrictions.

## Supplementary Materials

The following supporting information can be downloaded at: https://www.mdpi.com/article/10.3390/children13080989/s1. Table S1: Detailed overview of reported pediatric cases of metastatic neuroblastoma involving the mandible.

## Abbreviations

The following abbreviations are used in this manuscript:

AB	Antibiotic
ADX	Adrenalectomy
ALK	Anaplastic lymphoma kinase
ASCT	Autologous stem cell transplantation
bilat.	Bilateral
CR	Complete remission
CT	Computed tomography
CTx	Chemotherapy
d	Day(s)
dec.	Deceased
dx	Diagnosis
F	Female
FISH	Fluorescence in situ hybridization
FU	Follow-up
GD2	Disialoganglioside GD2
HVA	Homovanillic acid
INPC	International Neuroblastoma Pathology Classification
INRGSS	International Neuroblastoma Risk Group Staging System
INSS	International Neuroblastoma Staging System
IT	Immunotherapy
L	Left
LAD	Lymphadenopathy
M	Male
mand.	Mandibular
met.	Metastasis/metastatic
MIBG	Metaiodbenzylguanidine
mo	Month(s)
MPRAGE	Magnetization-prepared rapid gradient-echo
MRI	Magnetic resonance imaging
MYCN	MYCN proto-oncogene
NB	Neuroblastoma
NED	No evidence of disease
n.d.	Not determined
n.p.	Not possible
n.r.	Not reported
n.s.	Not specified
PICOST	Population, Intervention, Comparison, Outcome, Study design, Time
PRISMA	Preferred Reporting Items for Systematic Reviews and Meta-Analyses
R	Right
RT	Radiotherapy
SPECT/CT	Single-photon emission computed tomography/computed tomography
STIR	Short tau inversion recovery
TSE	Turbo spin-echo
Tx	Treatment/therapy
VMA	Vanillylmandelic acid
wk	Week(s)
y	Year(s)
